# Editorial: Revisit the roles of regulatory T cells in infection

**DOI:** 10.3389/fimmu.2022.1069344

**Published:** 2022-11-23

**Authors:** Xuyu Zhou, Jingxian Zhao, Bin Li, Xuguang Tai

**Affiliations:** ^1^ Chinese Academy of Sciences (CAS) Key Laboratory of Pathogen Microbiology and Immunology, Institute of Microbiology, Chinese Academy of Sciences (CAS), Beijing, China; ^2^ Savaid Medical School, University of Chinese Academy of Sciences, Beijing, China; ^3^ State Key Laboratory of Respiratory Diseases (SKLRD), National Clinical Research Centre for Respiratory Disease, Guangzhou Institute of Respiratory Health, the First Affiliated Hospital of Guangzhou Medical University, Guangzhou, China; ^4^ Guangzhou Laboratory, Guangzhou, Guangdong, China; ^5^ Shanghai Institute of Immunology, School of Medicine, Shanghai Jiao Tong University, Shanghai, China; ^6^ Experimental Immunology Branch, Center for Cancer Research, National Cancer Institute, National Institutes of Health (NIH), Bethesda, MD, United States

**Keywords:** infection, Treg, self, non-self, pathogen

Regulatory T cells (Tregs) are a unique CD4^+^ T cell subset expressing transcription factor Foxp3, which play a critical role in maintaining immunological self-tolerance. Similar to conventional T cells, Tregs have a highly diverse TCR repertoire that could recognize both self and non-self antigens. Engagement of TCR will trigger Treg activation allowing them to gain potent immune suppressive function and control immune homeostasis. Although Tregs are essential for preventing autoimmunity, their functionality in modulating the immune response against foreign antigens remains poorly defined. The suppressive activity of Tregs could have detrimental impacts on anti-pathogen responses, and overexpansion of Tregs often leads to the development of chronic infection. However, those suppressive effects have beneficial roles in controlling unfavorable immune responses in the mucosal system that is crucial for living in harmony with commensal bacteria. Moreover, in response to damage signals, Tregs might perform unique tissue-repairing functions in the local tissue, such as lung and muscle. A series of recent contributions in Frontiers in Immunology have discussed the potential role of Treg response for foreign antigens in various settings, including infections and vaccination.

The gastrointestinal tract is a large organ that contains a distinctive immune environment maintaining a delicate balance between immunological tolerance and activation. Billions of potentially pathogenic and commensal bacteria and enormous food antigens co-exist in the gut. Intestinal epithelial cells (IECs) form a physical barrier to segregate the external environment from the intestinal tissues to maintain tissue homeostasis. In the mini-review “*Reciprocal Interactions Between Regulatory T Cells and Intestinal Epithelial Cells*,” Jiang and Wu discussed the crosstalk between gut epithelium and resident Treg cells, including how IECs regulate intestinal Tregs induction and how Tregs counter-react with epithelium barrier to regulate its integrity.

The lung is another mucosal organ exposed to various potential pathogens through inhalation, and respiratory virus infections are still a significant public health problem that causes substantial morbidity globally. Many respiratory illnesses caused by virus infections share similar symptoms and are likely regulated by similar immunological mechanisms. Tregs are a critical peacekeeper during respiratory infection and influence the magnitude and severity of infections at different stages, which limit the early anti-viral response but suppress the inflammation caused by those infections, and are responsible for tissue repair. Xu et al. provided a comprehensive overview of clinical observations of the Treg dynamic during SARS-CoV-2 infection. Further, they discussed the potential of applying Tregs as adoptive therapy in severe COVID-19 patients. To gain more mechanistic insight into Tregs during lung infection, an original study by McGee et al. used the mouse influenza infection model to characterize the lung tissue-resident Treg response during primary and secondary influenza infections. Their work comprehensively describes lung resident Treg cells and highlights PD-1 and ICOS signaling in controlling tissue-resident Tregs expansion and IL-10 production.

The recent COVID-19 pandemic reveals again that aging is one of the most critical risk factors impacting the severity of many diseases. In the review article entitled “*The dark side of Tregs during aging*,” Palatella et al. summarized recent studies about qualitative as well as quantitative changes regarding Treg biology during aging and proposed that those age-related changes in Tregs could be an essential contributing factor for the increased vulnerability to a wide range of diseases observed in the elderly population. To address whether neonate infections impact the homeostatic maintenance of Tregs generated early in life. Yang et al. also provide an interesting study entitled “*Inflammatory perturbations in early life long-lastingly shape the transcriptome and TCR repertoire of the first wave of regulatory T cells.*” They demonstrated that even a single pathogenic stimulation during the neonatal stage could impact the first wave of Tregs.

This series of Research Topics provides an extensive update on the functionality of Treg in controlling the immune response against foreign antigens. Tregs are not simply suppressors for shutting down all immune responses. A recent Research Topic, “*Follicular Helper T Cells in Immunity and Autoimmunity*,” has also provided a comprehensive summary of TFRs, a population of Tregs expressing CXCR5 and PD-1, might play an essential helper role in the induction of optimal B cell responses. Some fundamental questions in this field are unanswered. Whether pathogen-specific Tregs could gain memory capacity after primary infection is a highly debated question. The microenvironment change following infection may also affect Treg identity and function; whether that is the missing link between infection and autoimmune disease is not clear. A deeper understanding of these molecular and cellular mechanisms would undoubtedly provide opportunities to treat various infectious diseases and benefit future vaccine development.

## Author contributions

All authors listed have made a substantial, direct, and intellectual contribution to the work and approved it for publication.

## Funding

This study has been supported by grants from the Ministry of Science and Technology of China Grants 2021YFC2300500 and the National Natural Science Foundation of China 81771694, 81971500. Our research is also supported by the National Key R&D Program of China 2019YFA09006100, National Natural Science Fund for Distinguished Young Scholars 31525008, and National Natural Science Foundation of China 32130041, 81830051 and 31961133011.

## Conflict of interest

Author BL is a co-founder of Biotheus Inc and chairman of its scientific advisory board.

The remaining authors declare that the research was conducted in the absence of any commercial or financial relationships that could be construed as a potential conflict of interest.

## Publisher’s note

All claims expressed in this article are solely those of the authors and do not necessarily represent those of their affiliated organizations, or those of the publisher, the editors and the reviewers. Any product that may be evaluated in this article, or claim that may be made by its manufacturer, is not guaranteed or endorsed by the publisher.

